# Une tumeur rare et distincte du cancer du sein: le carcinosarcome, à propos de huit cas et revue de la littérature

**DOI:** 10.11604/pamj.2013.14.127.689

**Published:** 2013-04-01

**Authors:** Samia Ghanem, Siham Khoyaali, Sara Naciri, Meriem Glaoui, Mohamed Mesmoudi, Hassan Errihani

**Affiliations:** 1 Service d'oncologie médicale, institut national d'oncologie médicale, Rabat, Maroc

**Keywords:** cancer du sein, carcinosarcome, métaplasique, HER2/neu, breast cancer, carcinosarcoma, metaplastic, HER2/neu

## Abstract

Le carcinosarcome du sein souvent appelé carcinome métaplasique du sein, est une tumeur maligne rare composée de deux lignées cellulaires distinctes, il est décrit comme un cancer du sein de type canalaire avec un composant de type sarcome. Il représente 0,08-0.2% de toutes les tumeurs malignes du sein. Il s'agit d'une étude rétrospective étalée sur un an, huit cas des carcinosarcomes mammaires ont été colligés à l'Institut national d'oncologie au Maroc durant l'année 2007. La médiane d’âge était de 49,5 ans, toutes les tumeurs étaient de haut grade, cliniquement 5 cas ont été classé t2 ou t3, et 3 cas classé sein localement avancé. Le traitement envisagé était basé sur une chirurgie mammaire suivie d'une radiothérapie et d'une chimiothérapie pour les cas adjuvants, l'envahissement ganglionnaire a été noté dans un cas, les récepteurs œstrogèniques sont négatifs, alors que les récepteurs progesteroniques sont positifs dans 4 cas, l'expression d'Her2 est absente dans tous les cas, le traitement des carcinosarcomes mammaires métastatiques était basé sur une chimiothérapie palliative. A 20 mois de médiane follow-up, la survie sans progression(SSP) pour le groupe entier est de 62,5%. Dans la limite de ce suivi, une rechute locorégionale a été détectée dans un cas, les deux patientes métastatiques sont décédées. Le carcinosarcome du sein est un sous-type rare du cancer du sein qui a un profil particulier et agressif, il a souvent un caractère triple négatif. Il est nécessaire de développer d'autres voies de recherche comme cibler le Récepteur HER1/EGFR.

## Introduction

Le carcinosarcome du sein, souvent appelé carcinome métaplasique du sein, est une tumeur maligne rare qui est composé de deux lignées cellulaires distinctes, il est décrit comme un cancer du sein de type canalaire avec une composante de type sarcome. Le carcinosarcome du sein est un cancer agressif, son pronostic est moins favorable par rapport aux autres types du cancer du sein qui sont plus fréquents comme le carcinome canalaire infiltrant ou lobulaire infiltrant. Le cancer du sein typique qui exprime les recépteurs hormonaux d'œstrogènes ou de progestérone répondent mieux au traitement hormonal et à la chimiothérapie. Les carcinomes métaplasiques du sein n′exprimant pas de façon générale les récepteurs d'œstrogène ou de progestérone et ne surexpriment pas l′oncogène HER2/neu, Ces tumeurs ont tendance à être plus agressifs en conséquence de ce phénotype «triple négatif ». Le facteur de croissance épidermique récepteur de protéine HER-1/EGFR est exprimé dans la majorité des carcinomes métaplasiques et peut donc potentiellement servir de cible thérapeutique pour les inhibiteurs de l′EGFR tels que le gefitinib et le cetuximab. A travers cette série de 8 cas, nous décrivons le caractère agressif de carcinosarcome du sein, nous renforçons les données de la littérature sur les caractéristiques de ces tumeurs qui n'expriment pas les récepteurs communs trouvés dans le cancer du sein typique. Ces rapports de cas soulignent également la nécessité d′enquêter sur le rôle du blocage du récepteur HER-1/EGFR avec des thérapies ciblées quand il est sur-exprimés dans la tumeur primitive.

## Méthodes

Huit (8) cas de carcinosarcomes (CS) primitifs du sein ont été colligé rétrospectivement à l'institut national d'oncologie Durant l'année 2007, toutes les patientes ont eu une mammographie et une échographie mammaire, avec un bilan d'extension: radiographie de poumons, échographie abdominale, dosage du CA15,3, le diagnostic histo-pathologique a été établie par biopsie ou par examen extemporané, l’étude immuno-histochimique a été réalisé, utilisant un panel EMA, cytokératines, desmin, vimentin, recherche des récepteurs d’‘estrogènes et de progestérone, recherche de protéine HER2/neu.

## Résultats

Huit (8) cas de carcinosarcomes du sein ont été colligés à l'institut national d'oncologie Durant l'année 2007 (Tableau 1). Toutes les tumeurs étaient de haut grade, la taille médiane était de 3.7 cm (range 1.4-9.5 cm). Le sein g est touché dans 4cas et le sein droit est touché dans 4cas, il n'y a pas de bilatéralité, cliniquement 5 cas ont été classé t2 ou t3, et 3 cas classés sein localement avancé, une mastectomie et un curage ganglionnaire ont été réalisé dans 5cas, un traitement conservateur a été opté dans un cas, l'envahissement ganglionnaire est noté dans un cas, un cas a eu une mastectomie dans le cadre d'une urgence pour une tumeur hémorragique.la moyenne du nombre de ganglions prélevés était de 12. Les limites de résection sont saines dans tous les cas, trois cas ont eu une radiothérapie adjuvante, la dose utilisée était 50gy sur la paroi thoracique ou sur le sein et dans un cas sur l'aire axillaire, deux cas était métastatique au niveau pulmonaire, les récepteurs ostrogéniques étaient négatifs dans tous les cas, 4 cas seulement exprimait les récepteurs à la progestérone. Le carcinome in situ était présent dans 3 cases. L'expression d'HER2/neu n'a été notée en aucun cas. A 20 mois de médiane follow-up, la survie sans progression(SSP) pour le groupe entier est de 62,5%.

**Tableau 1:** Caractéristiques cliniques, histopathologiques et thérapeutiques des carcinosarcomes du sein de notre série

Patiente	Age	Type histo	Grade	RE + RP	HER2	pT	pN	M	Chirurgie	CMT	RT	HORMONO	follow up	Etat
**1**	45	carcinosarc	2	RE = 0,RP = 80%	absent	t2	n0	0	conservateur	adj	oui	tam	45	bon
**2**	51	carcinosarc	3	RE = 0,RP = 0	absent	t3	n0	0	radical	adj + neoadj	non		37	bon
**3**	46	carcinosarc	3	RE = 0,RP = 0	absent	t3	n0	0	radical	adj	oui		5	rechute
**4**	77	metap	3	RE = 0,RP = 40%	absent	t2	n0	0	radical	adj	oui	tam	20	bon
**5**	54	metap	3	RE = 0,RP = 0	absent	t2	n1	0	radical	adj	oui		39	bon
**6**	48	metap	3	RE = 0,RP = 40%	absent	t4b	n0	1	radical	pall	non		8	décedée
**7**	59	metap	3	RE = 0,RP = 0	absent			1		pall	non		1	décedée
**8**	41	carcinosarc	2	RE = 0,RP = 80%	absent	t2	n0	0	radical	adj	oui	tam	20	bon

Type histo = type histopathologique, E= recepteurs oestrogéniques, RP= recepteurs progéstéroniques, pT: taille anatomopathologique, pN: classification ganglionnaire anatomopathologique, M: métastases, Conservateur = traitement chirurgical conservateur avec curage, radical= mastectomie radicale avec curage, CMT= chimiothérapie, adj = adjuvante, néoadj= néoadjuvante, pall = palliative, RT= radiothérapie adjuvante, tam = tamoxifène reçu

## Discussion

**Figure 1:**
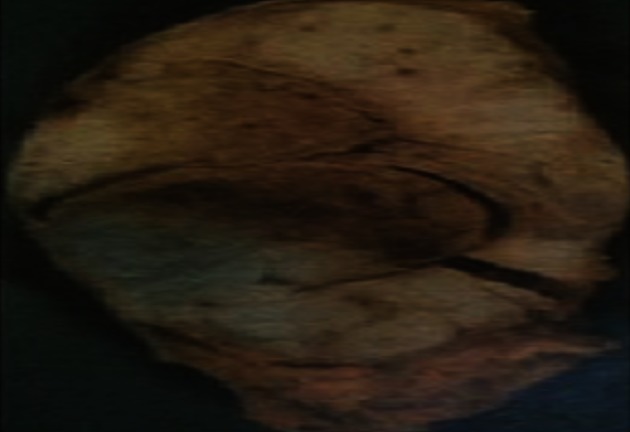
Tumeur de 16 cm, blanchâtre, polylobée

**Figure 2:**
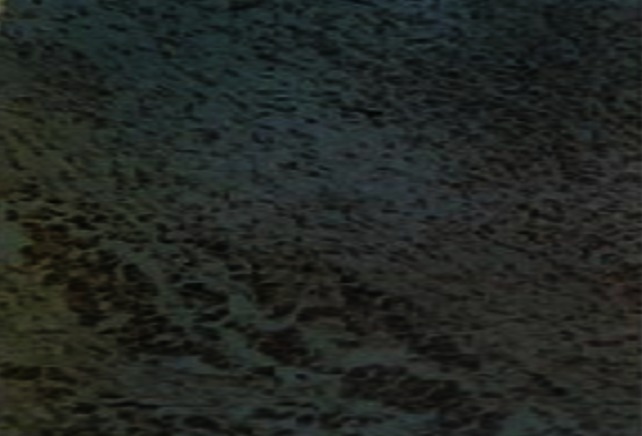
Carcinosarcome, double composantes carcinomateuse et sarcomateuse sans ligne de transition(Gx10)

**Figure 3:**
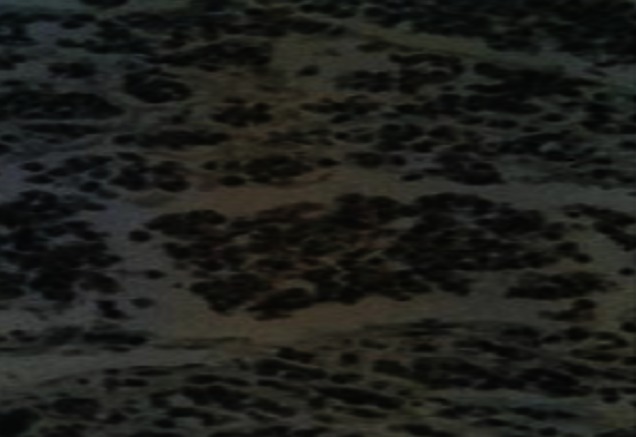
Composante carcinomateuse CK+ (Gx20)

**Figure 4:**
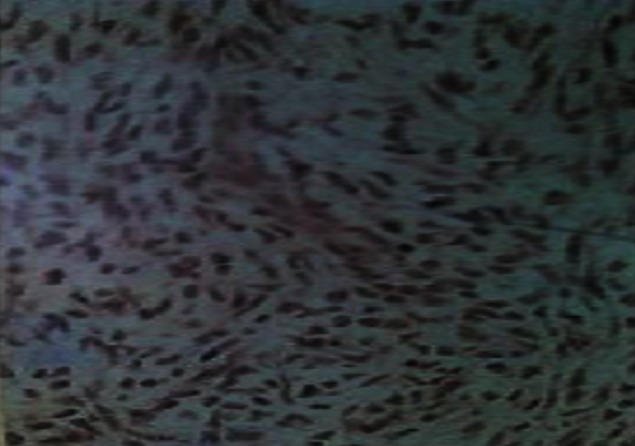
Composante sarcomateuse fibroblastique (Gx20)

Le carcinosarcome du sein (métaplasique, biphasique métaplasique, carcinoma métaplasique sarcomatoide, carcinoma sarcomatoide) est une tumeur rare, elle présente 0,08 à 0,2% de tous les cancers du sein [1, 2]. Le carcinosarcome a été obsérvé dans différents organes comme l'utérus, l'ovaire, le sein La vraie définition du carcinome métaplasique du sein (CMS) est une tumeur maligne du tissu épithélial (carcinome) mélangées avec des cellules malignes d′origine mésenchymateuse (sarcome) avec les caractéristiques cytologiques et histologiques identifiés sur les images de microscopie optique et sur les tests immunohistochimiques [2, 3]. En effet, ce type de tumeur est souvent appelé cancer du sein métaplasique, c'est une tumeur rare et inhabituelle qui est composé par un mélange de deux composants ou plus [4]. Le terme de carcinosarcome était utilisé pour les tumeurs où la ligne de transition entre les composantes carcinomateuse et sarcomateuse est bien respectée sur tous les champs microscopiques (Figure 1, Figure 2, Figure 3, Figure 4). Les cellules d′origine de cette tumeur ne sont pas encore déterminées, mais la plupart des recherches nous amène à croire que les cellules sont d'origines myoépithéliales. La composante tumorale peut être homogène adénosquameuse, ou hétérogène épithéliale (adénocarcinomes) et mésenchymateuse (matrice, à cellules fusiformes et sarcomateuse) [4, 5]. Wargotz et al [6] considère que les carcinosarcomes du sein (CSM) sont des carcinomes métaplasiques qui se développent à partir d'une seule cellule totipotente avec une différenciation biphasique [6–8]. Ils ont noté que la composante sarcomateuse du carcinosarcome exprime les marqueurs épithéliaux, cytokératines dans 55% des cas. Par contre l'expression d'actine et S-100 a été observées dans les deux composantes stromale et épithéliale dans 13% des cas. Cependant, il semble plus approprié d'employer le terme de carcinome métaplasique sarcomatoide pour designer cette entité du cancer du sein qui se présente avec les deux caractéristiques carcinomateuses et sarcomatoides [9]. Actuellement la connaissance du cancer du sein métaplasique est mieux établie vu la croissance de découverte du carcinome métaplasique du sein durant cette dernière décennie par rapport aux années précédentes.

La présentation clinique du cancer du sein métaplasique est identique à celle du carcinome canalaire invasif, les tumeurs du sein métaplasiques sont dans la plupart des cas peu différenciés, et de haut grade. Les récepteurs œstrogèniques (RO) et progestéroniques (RP) sont négatifs dans la majorité des cas et HER2-neu est négative par immunohistochimie [10]. Les résultats de notre série soulignent ces données de la littérature. Les RO sont négatifs pour les 8 cas, les RP sont positifs dans 4cas. Les caractéristiques cliniques et pathologiques du cancer du sein métaplasique sont importantes à distinguer des autres types peu fréquents du cancer du sein comme le carcinome à cellules fusiformes, fibrosarcome, ostéosarcome, histiocytome fibreux malin, tumeur phyllode et le sarcome stromal, la réponse au traitement de ces types ainsi que les taux de survie sont totalement différents du carcinome métaplasique du sein.

Hennessy et al. Ont rapporté une série de 100 cas de carcinome métaplasique biphasique (MSC) et 98 cas de carcinosarcome mammaire en 2005 [11]. Ils ont comparé les caractéristiques cliniques et les paramètres de survie de ces deux types de cancer [9] avec ceux de tous les cancers du sein traité au centre MD Anderson durant la même période. Ils concluent que ces deux entités MSC et carcinosarcome du sein sont des tumeurs agressives qui répondent mal au traitement et qu'ils ont des paramètres cliniques similaires et un pronostic identique au adénocarcinome peu différencié avec des récepteurs hormonaux négatifs. Ils ont constaté que la Taille initiale de la tumeur est un grand facteur prédicteur du pronostic. Les MSC et carcinosarcomes mammaires ont un taux élevé de rechute locorégionale plus particulièrement pour les stades T2 ou plus. Ce taux élevé de rechute locale doit inciter les chirurgiens à opter plus pour un traitement radical particulièrement avec les t2 ou plus.

Ces tumeurs sont généralement peu différencié avec des récepteurs hormonaux négatives et HER2 négatif, la réponse à la chimiothérapie néo adjuvante peut être efficace dans ce type de tumeurs puisqu'il existe une composante carcinomateuse, et que les CMS a beaucoup de similitudes avec carcinome mammaires mais il a aussi des caractéristiques « sarcoma-like » (ganglions non envahis, l'importance de la taille de la tumeur comme marqueur pronostic), une patiente de notre série a été traité par une chimiothérapie néoadjuvante à base d'anthracycline pendant 4 cycles avec une réponse partielle mais la tumeur est resté toujours ulcérobourgeonnante et purulente, la tumeur a été opéré dans le cadre d'une exérèse de propreté avec marges chirurgicales négatives et curage ganglionnaire négatif, suivie de chimiothérapie adjuvante, la patiente est resté en bon control jusqu’à présent avec un follow-up de 36 mois. Concernant le pronostic, Beatty et al ont rapporté une étude récente de 16 publications sur une période de 21 ans (1984-2005) qui étudie caractéristiques cliniques et pathologiques du CSM et qui a révélé une survie globale à 5 ans allant de 49 à 68% [4]. Toutefois, lorsque la survie globale à 5ans et la survie sans récidive ont été comparé à une cohorte de patients atteints de cancer du sein en général, la survie à 5 ans sans récidive et globale n′était pas significativement différentes (84% vs 93%, 83%, 90%, respectivement). Hennessy et al ont rapporté la SG à 5ans et SSP par rapport au stade de la maladie pour les CSM et CSM [11]. Pour 199 and 81 MSC et carcinosarcome, le taux de survie globale des patients qui ont une maladie localisée patients (stade I-III) est de 63% et 60 %, respectivement. La SSP à 5ans était de 72% et 73% respectivement. Pour nos patientes, 3 cas adjuvants sont resté en bon control, la taille tumorale était classé t2, on a noté une récidive locale du carcinosarcome chez une patiente peut être dû au fait que les 3 patientes ont eu un traitement chirurgical radical ou parce que le follow-up n'est pas assez suffisant, une patiente a présenté une récidive locorégionale après la fin de son traitement adjuvant, une patiente est perdue de vue après la fin de chimiothérapie adjuvante, une patiente a eu une exérèse de propreté parce que la tumeur était localement avancé, elle est actuellement en bon control de sa maladie avec un follow-up de 36 mois. Les métastases pulmonaires sont plus fréquentes que les métastases cérébrales, osseuses ou hépatiques, et le pronostic de ces patients métastatiques est faible [12]. deux patientes de notre série étaient métastatiques, une patiente était en mauvais état général et décédé en quelque jours après la chirurgie palliative, la deuxième patiente a progressé après 2cures de chimiothérapie puis décédé par la suite dans un contexte de détérioration de son état général ce qui souligne le profil agressif de ces tumeurs.

En général, les options thérapeutiques recommandées dans le traitement du carcinosarcome mammaire suivent les principes thérapeutiques pour le cancer du sein invasif. Dans la majorité des cas rapportés, la mastectomie avec ou sans curage ganglionnaire axillaire a été effectuée, suivie d′une chimiothérapie post-opératoire et de la radiothérapie.

Dans la majorité des carcinomes métaplasiques sarcomatoide du sein, il y'a une surexpression du récepteur HER1/EGFR. Dans une étude de 20 cas de carcinomes métaplasiques du sein, ils ont constaté que 14/20MSC ont été positifs pour l′expression de l′EGFR [13], sa recherche devraient être inclus dans l′évaluation initiale des carcinosarcomes mammaires. De nouvelles possibilités thérapeutiques ont été suggérées par le traitement par agents ciblant l′EGFR tels que le gefitinib (ZD1839, Iressa) et le cétuximab (Erbitux) dans une stratégie adjuvante. Les anticorps monoclonaux et les inhibiteurs de petites molécules de l′EGFR sont actuellement évalués chez les patients atteints de cancer du poumon. Il a été suggéré que l′expression de l′EGFR en l′absence de récepteurs hormonaux ou d′autres récepteurs de la famille EGFR pourrait rendre le carcinome métaphasique du sein encore plus sensible aux inhibiteurs de tyrosine kinase EGFR [14]. D′autres recherches devraient être effectuées afin d′évaluer l'efficacité d′un tel traitement ciblant l'EGFR dans le carcinome métaplasique du sein.

## Conclusion

Même si le carcinosarcome du sein est un sous type rare du cancer du sein, il a un intérêt important en raison de son hétérogénéité clinique et pathologique par rapport au cancer du sein typique. Il est important de souligner que le carcinome métaplasique et le carcinosarcome du sein semblent rarement surexprimé l'oncoprotéine HER2/neu. Les récepteurs oestrogénique et progestéronique sont négatifs dans la majorité des cas. La SSP faible associé aux carcinosarcomes du sein incite à développer les recherches cliniques et d'explorer le mécanisme de cancérogenèse de ce type de tumeur et notamment le ciblage du Récepteur HER1/EGFR pour un meilleur traitement de ce type de tumeur.
